# Checklist of bees (*Apoidea*) from a private conservation property in west-central Montana

**DOI:** 10.3897/BDJ.5.e11506

**Published:** 2017-03-30

**Authors:** Marirose Kuhlman, Skyler Burrows

**Affiliations:** 1 MPG Ranch, Florence, MT, United States of America; 2 Bee Biology and Systematics Lab, Logan, UT, United States of America

**Keywords:** Hymenoptera, biodiversity, pollinator, native species, invasive species, adventive, range expansion, introduced, *Megachile apicalis*, North America, Intermountain West

## Abstract

**Background:**

Here we present preliminary results from the first three years of a long-term bee survey conducted at a 3,840-ha private conservation property in the northern Sapphire Mountains and Bitterroot River Valley, and a pilot study at an associated 80-ha property in the Swan River Valley, Missoula County, Montana, USA. The survey includes hand-net, bowl-trap, and blue-vane trap collections. The resulting checklist comprises 229 bee species and morphospecies within 5 families, 38 genera and 91 subgenera. Of the total species in the list, 34 of them represent first state records Montana. This survey expands the number of bee species recorded in Montana to 366. Included in these species is Megachile (Eutricharaea) apicalis Spinola, showing a range expansion for this introduced bee.

**New information:**

We present new distributional records for 34 bee species, including Megachile (Eutricharaea) apicalis Spinola, an introduced bee that was discovered to be resident in North America in 1984 in Santa Barbara County, California. This species has since expanded its range in the across the west, but had not been previously recorded in Montana.

## Introduction

Wild bees play a vital role as pollinators in both agricultural and natural systems, and may be important to the success of habitat restoration projects (Menz et al. 2011, Williams 2011). Work has been done to document bee faunas of some western regions of North America, including Colorado (Scott et al. 2011), the Columbia Basin (Tepedino and Griswold 1995), north-central Washington (Wilson et al. 2010), and some US national parks and monuments (e.g. Messinger and Griswold 2002); but there are few large-scale bee surveys from Montana.

Reported here is the bee species list from the first three years of a long-term bee monitoring study we initiated in 2013 at MPG Ranch, a privately-owned conservation property in the northern Bitterroot Valley and Sapphire Mountains, and a pilot study from MPG North, an associated property in the Swan Valley. Both properties are in Missoula County of west-central Montana. A purpose of this study is to inventory the bee fauna on the properties.

## Materials and methods

### Study Site

**Figure 1.**
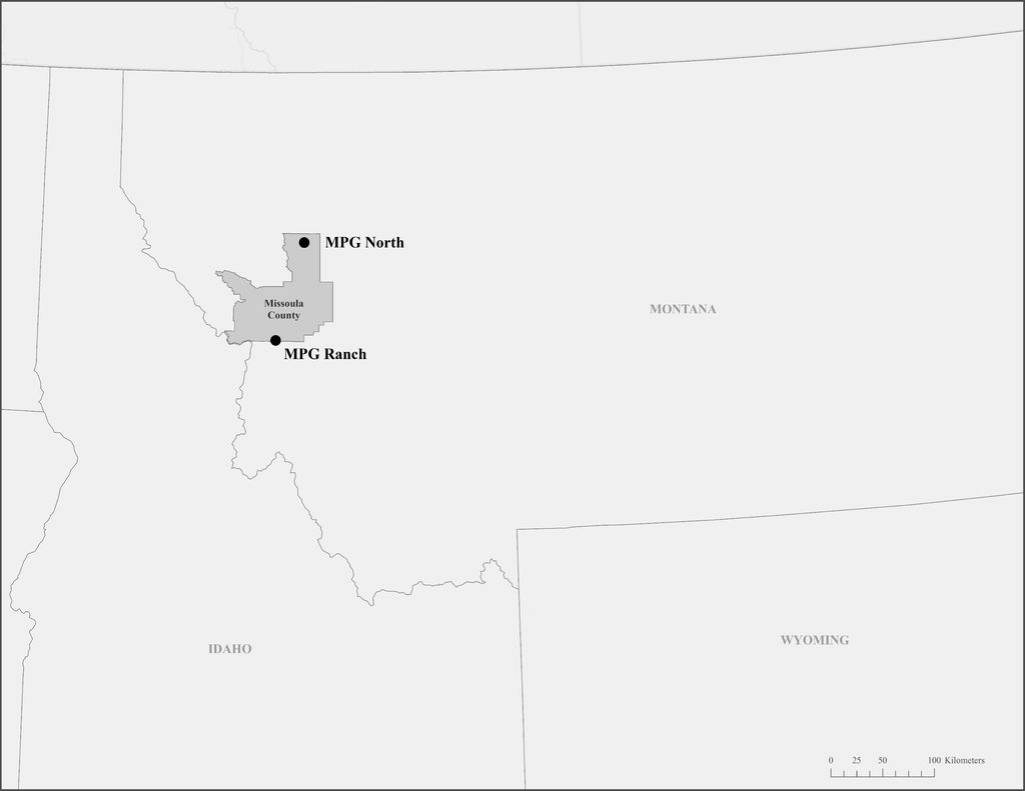
Map of Montana and surrounding states with Missoula County boundary and property locations marked.

The 3,840 hectare MPG Ranch is a privately-owned conservation property in west-central Montana, in the Bitterroot River Valley and Sapphire Mountains of Missoula County (Fig. 1). Elevations range from 975m at the valley floor along the Bitterroot River, to 1860m on Mount Baldy, its highest point. The region has a mild, semi-arid climate with cool, moist winters and warm, dry summers. Mean precipitation ranges from 300mm at the valley floor to 350mm on mountain summits; most precipitation occurs in the winter as snow. Mean temperatures for the nearby town of Stevensville range from -3.6°C in December to 20.0°C in July.

For over a century prior to 2009, this property had been managed for livestock and agricultural crop production. During that time, most of the lower elevation grasslands were replaced with irrigated crops or introduced forage grasses. These areas are now out of production and undergoing restoration treatments to return them to a more natural state. Livestock has also been removed from the property as part of the restoration effort, and native ungulates (elk, mule deer, and whitetail deer) currently comprise the majority of vertebrate grazers. Habitats on the property include riparian bottomlands, dry open forests, mid-elevation sagebrush steppe and grasslands, montane grasslands, and montane mixed-conifer forest.

The MPG North, an associated 81 hectare conservation property separated from MPG Ranch by over 100 kilometers, is located in the Swan River Valley, also in Missoula County (Fig. 1), at an elevation of 1200m. The climate in the Swan River Valley is cooler and wetter than the Bitterroot River Valley. At the nearby town of Seeley Lake, mean temperatures range from -6.1°C in December to 17.5°C in July, and mean annual preciptiation is 530mm. MPG North's habitat consists of mixed-conifer forest and clearings, and some small wetlands.

### Collection Methods

We captured bees from MPG Ranch using the protocol outlined in *A Standardized Method for Monitoring Bee Populations: The Bee Inventory (BI) Plot* (LeBuhn et al. 2003), modified by reducing the amount of time spent netting from 60 minutes to 30 minutes. We used 24 plots that had been chosen for a larger, multi-organism surveying effort occurring on the property. These 24 sampling plots were placed along an elevational gradient, from approximately 975m elevation to 1850m elevation. At each location we laid out two 50m transects, intersecting at 25m, creating an "X". We deployed 21, 3.25oz Solo brand plastic cups (Solo brand stock number PB6-0099) filled with soapy water at each sampling plot and placed them about 5m apart along the transects. Bowls were spray-painted fluorescent blue, fluorescent yellow, or left white. Seven bowls of each of the three colors were used at each site. With the exception of the first sampling round, we placed traps out at all sampling plots on the same day by 9am and collected the contents after 3pm, providing a snapshot of the bee species composition for that day. We seived bowl-trapped bees in the field and transferred them into 4oz Whirl-Pak bags with 70% isopropanol. We kept these samples refrigerated until shipment to the USDA-ARS Pollinating Insects Research Unit in Logan, UT, for identification. A total of 30 minutes of netting occurred at each site on each collection day in 2013 and 2015, when time and field staff availability permitted. Netting took place on bowl-trapping days or the following day, and was limited to a 100m radius of plot center. Samplers netted bees throughout the plot and attempted to sample from as many different floral resources as possible within the time frame. Netted bees were dispatched with ethyl acetate and kept frozen in labeled collection tubes until processed.

We sampled every 2-4 weeks throughout as much of the field season as possible to capture as much of the bee fauna as we could. We sampled each of the plots five times in 2013 (June 10-17, July 1, July 22, August 12, and September 9), seven times in 2014 (May 1, May 15, June 12, July 10, July 31, August 28, and September 17), and nine times in 2015 (April 21, May 4, May 18, June 8, June 29, July 20, August 10, September 1, and September 23).

Collecting events at MPG North were part of a pilot study and consisted of a small number of blue vane trap collections and some hand-netting in 2014 and 2015. Bee species caught at MPG North are included in this species checklist.

Species accumulation estimates were determined by using EstimateS (Colwell 2013).

### Species Identification

Bees were identified by Skyler Burrows with help from Harold Ikerd, Zachary Portman, Michael Orr, and Terry Griswold. Family, genus, and subgenus classifications follow Michener 2007 except in the some of the Lasioglossum subgenera (which follow Gibbs et al. 2012). Species determinations were made using the following taxonomic keys: Colletidae (Snelling 1966a, Snelling 1966b, Snelling 1970, Stephen 1954), Andrenidae (Bouseman and LaBerge 1978, LaBerge and Bouseman 1970, LaBerge 1969, LaBerge 1973, LaBerge and Ribble 1975, LaBerge 1977, LaBerge 1980, LaBerge 1985, LaBerge 1986, LaBerge 1989, Ribble 1967, Ribble 1974, Thorp 1969, Timberlake 1956, Timberlake 1960) Halictidae (Dumesh and Sheffield 2012, McGinley 1986, McGinley 2003, Roberts 1972, Roberts 1973), Megachilidae (Baker 1975, Gonzalez and Griswold 2013, Grigarick and Stange 1968, Hurd and Michener 1955, Michener 1938a, Michener 1938b, Michener 1939, Hurd 1958, Michener 1947, Sandhouse 1939, Sheffield et al. 2011), Apidae (Brumley 1965, Daly 1973, Koch et al. 2012, Hurd and Linsley 1951, LaBerge 1956a, LaBerge 1956b, LaBerge 1961, Laberge 1963, Rightmyer 2008, Sipes 2001, Thorp and Horning 1983, Timberlake 1969). In addition, reference specimens from the U.S. National Pollinating Insect Collection were used for species verification and identification.

Bees that could be morphologically distinguished from each other but lacked taxonomic literature to determine species level were separated into morphospecies and given alphanumeric titles. When male and female morphospecies could not be associated, male morphospecies were given letters (e.g Nomada sp. A) and female morphospecies were given numbers (eg. Lasioglossum sp. 01). We only included the gender with the larger number of morphospecies in our species counts to avoid falsely inflating the number of species.

Bee specimens were pinned and labeled with location information, date, collection method, and collector name. Except for two synoptic sets of voucher specimens that are kept at MPG Ranch, specimens are deposited in the U.S. National Pollinating Insect Collection at Logan, Utah.

*Lasioglossum* bees in the subgenus *Dialictus* were identified only to subgenus due to the difficulty in distinguishing between *Dialictus* species and the lack of comprehensive keys for the western United States. Species in the genus *Sphecodes* were also only identified to genus level due to the lack of available taxonomic literature for the area.

### Range

Species ranges were determined using Global Biodiversity Information Facility (GBIF), DiscoverLife.org (Ascher and Pickering 2016), and U.S. National Pollinating Insects databases in order to determine how many species records were new to the state of Montana.

## Checklists

### Checklist

#### Colletidae

#### Colletes consors consors

Cresson 1868

#### Colletes phaceliae

Cockerell 1906

#### Colletes fulgidus fulgidus

Swenk 1904

#### Colletes kincaidii

Cockerell 1898

#### Hylaeus (Hylaeus) annulatus

(Linnaeus 1758)

#### Hylaeus (Hylaeus) conspicuus

(Metz 1911)

#### Hylaeus (Hylaeus) mesillae

(Cockerell 1896)

#### Hylaeus (Paraprosopis) coloradensis

(Cockerell 1896)

#### Hylaeus (Paraprosopis) wootoni

(Cockerell 1896)

#### Hylaeus (Prosopis) modestus citrinifrons

(Cockerell 1896)

#### Andrenidae

#### Andrena (Andrena) milwaukeensis

Graenicher 1903

#### Andrena (Andrena) schuhi

LaBerge 1980

##### Notes

New species record for Montana

#### Andrena (Andrena) thaspii

Graenicher 1903

#### Andrena (Andrena) vicinoides

Viereck 1904

#### Andrena (Andrena) sp. A

#### Andrena (Diandrena) evoluta

Linsley & MacSwain 1961

##### Notes

New species record for Montana

#### Andrena (Euandrena) astragali

Viereck & Cockerell 1914

#### Andrena (Euandrena) lawrencei

Viereck & Cockerell 1914

#### Andrena (Euandrena) nigrihirta

(Ashmead 1890)

#### Andrena (Euandrena) nigrocaerulea

Cockerell 1897

#### Andrena (Holandrena) cressonii

Robertson 1891

#### Andrena (Leucandrena) barbilabris

(Kirby 1802)

#### Andrena (Melandrena) nivalis

Smith 1853

#### Andrena (Melandrena) transnigra

Viereck 1904

#### Andrena (Melandrena) vicina

Smith 1853

#### Andrena (Micrandrena) chlorogaster

Viereck 1904

##### Notes

New species record for Montana

#### Andrena (Micrandrena) illinoiensis

Robertson 1891

#### Andrena (Micrandrena) melanochroa

Cockerell 1898

##### Notes

New species record for Montana

#### Andrena (Micrandrena) microchlora

Cockerell 1922

#### Andrena (Micrandrena) piperi

Viereck 1904

##### Notes

New species record for Montana

#### Andrena (Micrandrena) cf. robinsoni

Lanham 1987

#### Andrena (Plastandrena) crataegi

Robertson 1893

#### Andrena (Plastandrena) prunorum

Cockerell 1896

#### Andrena (Scaphandrena) merriami

Cockerell 1901

#### Andrena (Scaphandrena) scurra

Viereck 1904

#### Andrena (Scaphandrena) sladeni

Viereck 1924

##### Notes

New species record for Montana

#### Andrena (Simandrena) angustitarsata

Viereck 1904

#### Andrena (Simandrena) pallidifovea

(Viereck 1904)

#### Andrena (Thysandrena) candida

Smith 1879

#### Andrena (Thysandrena) medionitens

Cockerell 1902

#### Andrena (Thysandrena) w-scripta

Viereck 1904

#### Andrena (Trachandrena) amphibola

(Viereck 1904)

#### Andrena (Trachandrena) cupreotincta

Cockerell 1901

#### Andrena (Trachandrena) forbesii

Robertson 1891

#### Andrena (Trachandrena) miranda

Smith 1879

#### Andrena (Trachandrena) salicifloris

Cockerell 1897

#### Andrena (Tylandrena) subtilis

Smith 1879

#### Calliopsis xenus

(Rozen 1958)

##### Notes

New species record for Montana

#### Panurginus atriceps

(Cresson 1878)

#### Panurginus sp. 01

#### Perdita (Perdita) fallax

Cockerell 1896

#### Perdita (Perdita) swenki

Crawford 1915

##### Notes

New species record for Montana

#### Perdita (Pygoperdita) wyomingensis segona

Timberlake 1956

#### Pseudopanurgus didirupa

(Cockerell 1908)

##### Notes

New species record for Montana

#### Halictidae

#### Agapostemon (Agapostemon) obliquus

(Provancher 1888)

##### Notes

New species record for Montana

#### Agapostemon (Agapostemon) texanus

Cresson 1872

#### Agapostemon (Agapostemon) virescens

(Fabricius 1775)

#### Dufourea holocyanea

(Cockerell 1925)

#### Dufourea maura

(Cresson 1878)

#### Dufourea trochantera

Bohart 1948

#### Halictus (Nealictus) farinosus

Smith 1853

#### Halictus (Odontalictus) ligatus

Say 1837

#### Halictus (Protohalictus) rubicundus

(Christ 1791)

#### Halictus (Seladonia) confusus

Smith 1853

#### Halictus (Seladonia) tripartitus

Cockerell 1895

#### Lasioglossum (Dialictus) sp.

#### Lasioglossum (Hemihalictus) glabriventre

(Crawford 1907)

#### Lasioglossum (Hemihalictus) inconditum

(Cockerell 1916)

#### Lasioglossum (Hemihalictus) kincaidii

(Cockerell 1898)

##### Notes

New species record for Montana

#### Lasioglossum (Hemihalictus) ovaliceps

(Cockerell 1898)

##### Notes

New species record for Montana

#### Lasioglossum (Hemihalictus) ruficorne

(Crawford 1907)

##### Notes

New species record for Montana

#### Lasioglossum (Hemihalictus) sp. 1

#### Lasioglossum (Hemihalictus) sp. 2

#### Lasioglossum (Hemihalictus) sp. 3

#### Lasioglossum (Hemihalictus) sp. 4

#### Lasioglossum (Lasioglossum) anhypops

McGinley 1986

#### Lasioglossum (Lasioglossum) athabascense

(Sandhouse 1933)

#### Lasioglossum (Lasioglossum) egregium

(Vachal 1904)

#### Lasioglossum (Lasioglossum) paraforbesii

McGinley 1986

#### Lasioglossum (Lasioglossum) sisymbrii

(Cockerell 1895)

#### Lasioglossum (Lasioglossum) trizonatum

(Cresson 1874)

#### Lasioglossum (Lasioglossum) zonulum

(Smith 1848)

#### Lasioglossum (Sphecodogastra) cooleyi

(Crawford 1906)

#### Lasioglossum (Sphecodogastra) lusoria

(Cresson 1872)

#### Sphecodes sp.

#### Megachilidae

#### Anthidium (Anthidium) atrifrons

Cresson 1868

#### Anthidium (Anthidium) mormonum

Cresson 1878

#### Anthidium (Anthidium) utahense

Swenk 1914

#### Ashmeadiella (Ashmeadiella) bucconis

(Say 1837)

#### Ashmeadiella (Ashmeadiella) cactorum

(Cockerell 1897)

#### Ashmeadiella (Ashmeadiella) californica

(Ashmead 1897)

##### Notes

New species record for Montana

#### Ashmeadiella (Ashmeadiella) cubiceps

(Cresson 1879)

##### Notes

New species record for Montana

#### Atoposmia (Atoposmia) elongata

(Michener 1936)

#### Chelostoma (Chelostoma) minutum

Crawford 1916

##### Notes

New species record for Montana

#### Coelioxys (Boreocoelioxys) banksi

Crawford 1914

#### Coelioxys (Boreocoelioxys) moesta

Cresson 1864

#### Coelioxys (Boreocoelioxys) octodentata

Say 1824

#### Coelioxys (Boreocoelioxys) rufitarsis

Smith 1854

#### Coelioxys (Cyrtocoelioxys) modesta

Smith 1854

#### Coelioxys (Xerocoelioxys) edita

Cresson 1872

##### Notes

New species record for Montana

#### Coelioxys (Xerocoelioxys) grindeliae

Cockerell 1900

#### Dianthidium (Dianthidium) curvatum

(Smith 1854)

#### Dianthidium (Dianthidium) pudicum

(Cresson 1879)

#### Dianthidium (Dianthidium) subparvum

Swenk 1914

#### Dianthidium (Dianthidium) ulkei

(Cresson 1878)

#### Dioxys pomonae

Cockerell 1910

##### Notes

New species record for Montana

#### Heriades (Neotrypetes) carinatus

Cresson 1864

#### Heriades (Neotrypetes) variolosus

(Cresson 1872)

#### Hoplitis (Alcidamea) grinnelli

Cockerell 1910

##### Notes

New species record for Montana

#### Hoplitis (Alcidamea) pilosifrons

(Cresson 1864)

#### Hoplitis (Alcidamea) producta

(Cresson 1864)

#### Hoplitis (Alcidamea) sambuci

Titus 1904

##### Notes

New species record for Montana

#### Hoplitis (Cyrtosmia) hypocrita

(Cockerell 1906)

#### Hoplitis (Monumetha) albifrons

(Kirby 1837)

#### Hoplitis (Monumetha) fulgida fulgida

(Cresson 1864)

#### Megachile (Argyropile) parallela

Smith 1853

#### Megachile (Chelostomoides) angelarum

Cockerell 1902

##### Notes

New species record for Montana

#### Megachile (Eutricharaea) apicalis

Spinola 1808

##### Notes

New species record for Montana

#### Megachile (Eutricharaea) rotundata

(Fabricius 1793)

#### Megachile (Litomegachile) brevis

Say 1837

#### Megachile (Litomegachile) coquilletti

Cockerell 1915

##### Notes

New species record for Montana

#### Megachile (Litomegachile) lippiae

Cockerell 1900

#### Megachile (Litomegachile) onobrychidis

Cockerell 1905

#### Megachile (Megachile) lapponica

Thomson 1872

#### Megachile (Megachile) montivaga

Cresson 1878

#### Megachile (Megachile) relativa

Cresson 1878

#### Megachile (Megachiloides) pascoensis

Mitchell 1934

#### Megachile (Sayapis) fidelis

Cresson 1878

#### Megachile (Sayapis) pugnata

Say 1837

#### Megachile (Xanthosarus) melanophaea

Smith 1853

#### Megachile (Xanthosarus) perihirta

Cockerell 1898

#### Osmia (Acanthosmioides) integra

Cresson 1878

#### Osmia (Acanthosmioides) longula

Cresson 1864

#### Osmia (Cephalosmia) californica

Cresson 1864

#### Osmia (Cephalosmia) marginipennis

Cresson 1878

#### Osmia (Cephalosmia) montana montana

Cresson 1864

#### Osmia (Helicosmia) coloradensis

Cresson 1878

#### Osmia (Melanosmia) albolateralis

Cockerell 1906

#### Osmia (Melanosmia) atrocyanea

Cockerell 1897

#### Osmia (Melanosmia) austromaritima

Michener 1936

##### Notes

New species record for Montana

#### Osmia (Melanosmia) brevis

Cresson 1864

#### Osmia (Melanosmia) bruneri

Cockerell 1897

#### Osmia (Melanosmia) calla

Cockerell 1897

#### Osmia (Melanosmia) cyaneonitens

Cockerell 1906

#### Osmia (Melanosmia) densa

Cresson 1864

#### Osmia (Melanosmia) ednae

Cockerell 1907

#### Osmia (Melanosmia) juxta

Cresson 1864

#### Osmia (Melanosmia) kincaidii

Cockerell 1897

##### Notes

New species record for Montana

#### Osmia (Melanosmia) melanopleura

Cockerell 1916

##### Notes

New species record for Montana

#### Osmia (Melanosmia) paradisica

Sandhouse 1924

#### Osmia (Melanosmia) proxima

Cresson 1864

#### Osmia (Melanosmia) pusilla

Cresson 1864

#### Osmia (Melanosmia) trevoris

Cockerell 1897

#### Osmia (Melanosmia) tristella

Cockerell 1897

#### Osmia (Mystacosmia) nemoris

Sandhouse 1924

#### Osmia (Osmia) lignaria propinqua

Cresson 1864

#### Stelis (Stelis) Ashmeadiellae

Timberlake 1941

#### Stelis (Stelis) holocyanea

(Cockerell 1925)

#### Stelis (Stelis) lateralis

Cresson 1864

#### Stelis (Stelis) montana

Cresson 1864

#### Stelis (Stelis) sp. 01

#### Apidae

#### Anthophora (Clisodon) terminalis

Cresson 1869

#### Anthophora (Lophanthophora) pacifica

Cresson 1878

##### Notes

New species record for Montana

#### Anthophora (Lophanthophora) porterae

Cockerell 1900

#### Anthophora (Lophanthophora) ursina

Cresson 1869

#### Anthophora (Melea) bomboides

Kirby 1838

#### Anthophora (Melea) occidentalis

Cresson 1869

#### Anthophora (Mystacanthophora) urbana

Cresson 1878

#### Anthophora (Mystacanthophora) walshii

Cresson 1869

##### Notes

New species record for Montana

#### Apis mellifera

Linnaeus 1758

#### Bombus (Bombias) nevadensis

Cresson 1874

#### Bombus (Bombus) occidentalis

Greene 1858

#### Bombus (Cullumanobombus) rufocinctus

Cresson 1863

#### Bombus (Fervidobombus) californicus

Smith 1854

#### Bombus (Fervidobombus) fervidus

(Fabricius 1798)

#### Bombus (Psithyrus) fernaldae

(Franklin 1911)

#### Bombus (Psithyrus) insularis

(Smith 1861)

#### Bombus (Pyrobombus) bifarius

Cresson 1878

#### Bombus (Pyrobombus) centralis

Cresson 1864

#### Bombus (Pyrobombus) flavifrons

Cresson 1863

#### Bombus (Pyrobombus) huntii

Greene 1860

#### Bombus (Pyrobombus) mixtus

Cresson 1878

#### Bombus (Pyrobombus) vagans

Smith 1854

#### Bombus (Subterraneobombus) appositus

Cresson 1878

#### Ceratina (Zadontomerus) acantha

Provancher 1895

##### Notes

New species record for Montana

#### Ceratina (Zadontomerus) nanula

Cockerell 1897

#### Ceratina (Zadontomerus) neomexicana

Cockerell 1901

#### Diadasia diminuta

(Cresson 1878)

#### Epeolus americanus

(Cresson 1878)

#### Epeolus compactus

Cresson 1878

#### Epeolus minimus

(Robertson 1902)

##### Notes

New species record for Montana

#### Epeolus sp. 01

#### Eucera (Synhalonia) edwardsii

(Cresson 1878)

#### Eucera (Synhalonia) frater lata

(Provancher 1888)

#### Eucera (Synhalonia) fulvitarsis

(Cresson 1878)

#### Eucera (Synhalonia) hurdi

(Timberlake 1969)

##### Notes

New species record for Montana

#### Habropoda cineraria

(Smith 1879)

#### Melecta (Melecta) pacifica

Cresson 1878

#### Melecta (Melecta) separata

Cresson 1879

#### Melecta (Melecta) thoracica

Cresson 1875

##### Notes

New species record for Montana

#### Melissodes (Callimelissodes) composita

Tucker 1909

#### Melissodes (Callimelissodes) lupina

Cresson 1878

#### Melissodes (Callimelissodes) sp. 1

#### Melissodes (Eumelissodes) agilis

Cresson 1878

#### Melissodes (Eumelissodes) microsticta

Cockerell 1905

#### Melissodes (Eumelissodes) sp. 2

#### Melissodes (Heliomelissodes) rivalis

Cresson 1872

#### Neopasites (Neopasites) fulviventris

(Cresson 1873)

##### Notes

New species record for Montana

#### Nomada sp. 01

#### Nomada sp. 02

#### Nomada sp. 03

#### Nomada sp. 04

#### Nomada sp. 05

#### Nomada sp. 06

#### Nomada sp. 07

#### Nomada sp. 08

#### Nomada sp. 09

#### Nomada sp. 10

#### Nomada sp. 11

#### Nomada sp. 12

#### Nomada sp. A

#### Nomada sp. B

#### Nomada sp. C

#### Nomada sp. D

#### Nomada sp. E

#### Nomada sp. F

#### Nomada sp. G

#### Nomada sp. H

#### Nomada sp. I

#### Nomada sp. J

#### Nomada sp. K

#### Nomada sp. L

#### Nomada sp. M

#### Nomada sp. N

#### Nomada sp. O

#### Nomada scita

Cresson 1878

#### Triepeolus heterurus

(Cockerell & Sandhouse 1924)

##### Notes

New species record for Montana

#### Triepeolus micropygius

Robertson 1903

#### Triepeolus paenepectoralis

(Viereck 1905)

#### Triepeolus sp. 01

#### Xeromelecta (Melectomorpha) californica

(Cresson 1878)

## Analysis

**Figure 2.**
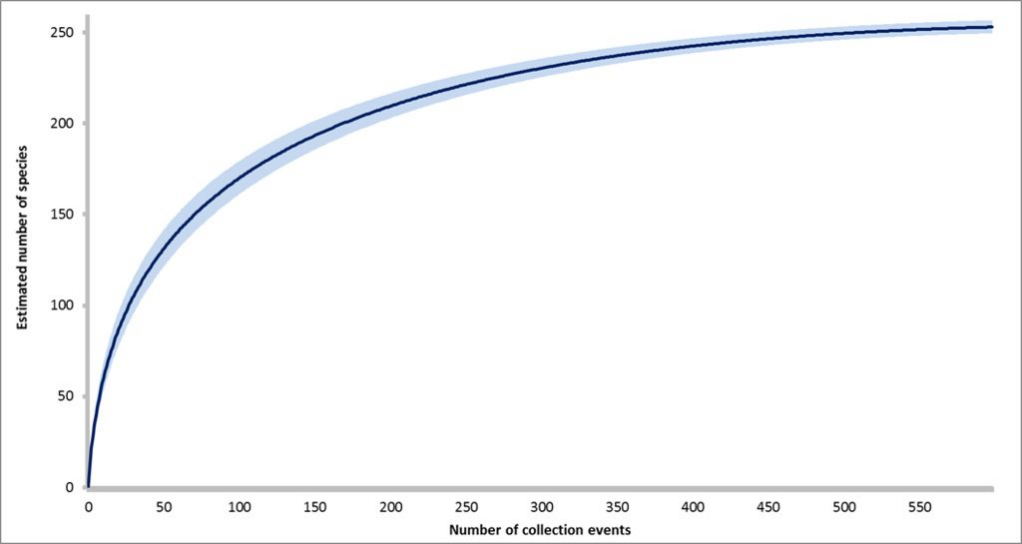
Species accumulation curve generated for bee species sampled in 558 collection events between 2013 and 2015. Species estimates were generated using EstimateS. The blue line represents the mean species accumulation, and the light blue shaded area represents the upper and lower bound of 95% confidence interval for species estimate.

Between 2013 and 2015 we collected a total of 64,747 bees representing 229 species and morphospecies across 38 genera in 5 bee families (Suppl. material 1). We had a total of 558 collection events (unique combinations of site and collection date). The number of species we collected is near the number of species we would expect to find in our study area based on species accumulation estimates, which predict the number of bee species in the area to be 255 (Fig. 2). Net collections yielded a total of 110 species, only three of which were species not also collected by pantraps. This number is lower than would be expected based on previous studies showing from 23% and 53% of the bee species collected by net to be unique when compared to pantraps (Grundel et al. 2011, Roulston et al. 2007, Wilson et al. 2008). This may be due to the reduced net collection time period (30 minutes instead of 60 minutes). In addition, many of the net collectors had no prior experience with insect collecting. Increased collection periods and improved collector training could raise the number of unique net collected species.

## Discussion

Prior to our investigation, the number of known wild bee species in Montana was 337. Our study increases this number to a total of 366. This result may not be unexpected since Montana is largely rural and there have been few large scale bee inventories in the state. Fewer than 1200 collected bee specimens have been reported each year in Montana prior to 2013 (GBIF 2016, U. S. National Pollinating Insects Database 2016). Our species list is relevant to other areas within the Montana Valley and Foothill Prairies Ecoregion (Taylor 2012), but may not reflect bee species present in other ecoregions in Montana, especially the Northwestern Great Plains and Northwestern Glaciated Plains Ecoregions. Investigations in the sparsely populated central and eastern portions of Montana may yield more species to a state bee species checklist.

### *Megachile apicalis*

*Megachile apicalis* is an Old World species in the subgenus Eutricharaea. Although there are North American records for *M. apicalis* prior to 1932, no established populations were recorded until 1984, when resident populations were documented in Santa Barbara County, CA (Cooper 1984). Subsquent collections showed that the species had expanded its range to Oregon, Washington, and British Columbia, Canada, along with its most common floral host, *Centaurea solstitialis* (Barthell et al. 2001, McIver et al. 2009, Sheffield et al. 2011), a widespread, introduced weed that invades rangelands in the western United States. There is evidence for a positive link between the spread of non-native weeds and the presence of non-native bees (Hanley and Goulson 2003). *Megachile apicalis* has a strong association with *C. solstitialis* and some researchers have suggested that these two Old World species, along with *Apis mellifera*, form an invasive mutualism (Barthell et al. 2001, McIver et al. 2009), in which each of the three species within the mutualism benefits from the presence of, and encourages the spread of the other species within the mutualism. Our collections are the first documentation of *M. apicalis* in Montana, indicating further range expansion of this adventive bee. *Centaurea stoebe*, a close relative of *C. solstitialis*, is a problematic weed species throughout MPG Ranch and Montana. *Megachile apicalis* captures increase in late summer/early autumn, when *Centaurea stoebe* bloom intensity peaks (MPG Ranch unpublished data), suggesting that *M. apicalis* may be preferentially using this *Centaurea* species in our area. Additionally, *M. apicalis* appears to be integrated throughout the MPG Ranch, since we have found *M. apicalis* at most of our sampling locations.

## Supplementary Material

Supplementary material 1 MPG species with male and female counts Data type: Occurence
Brief description: MPG species list with number of males and females of each species collected from 2013-2015.
File: oo_125429.xlsx Burrows, S.J. & Kuhlman M.
